# An experimental model of colitis induced by dextran sulfate sodium from acute progresses to chronicity in C57BL/6: correlation between conditions of mice and the environment 

**Published:** 2016

**Authors:** Niloofar Taghipour, Mahsa Molaei, Nariman Mosaffa, Mohammad Rostami-Nejad, Hamid Asadzadeh Aghdaei, Ali Anissian, Pedram Azimzadeh, Mohammad Reza Zali

**Affiliations:** 1 *Gastroenterology and Liver Diseases Research Center, Research Institute for Gastroenterology and Liver Diseases, Shahid Beheshti University of Medical Sciences, Tehran, Iran*; 2 *Basic and Molecular Epidemiology of Gastrointestinal Disorders Research Center, Research Institute for Gastroenterology and Liver Diseases, Shahid Beheshti University of Medical Sciences, Tehran, Iran*; 3 *Immunology Department, Faculty of Medicine,Shahid Beheshti University of Medical Sciences, Tehran, Iran*; 4 *Department of veterinary, College of Agriculture, Islamic Azad University, Abhar branch, Abhar, Iran*

**Keywords:** Inflammatory bowel disease, Murine models, DSS, C57BL/6

## Abstract

**Aim:**

To induce acute colitis progresses to chronicity in C57BL/6 mice by dextran sulfate sodium.

**Background:**

Murine models are essential tools to understand IBD pathogenesis. Among different types of chemically induced colitis models, the dextran sulfate sodium (DSS)-induced colitis model is the most common model of IBD, due to its simplicity.

**Patients and methods:**

Male C57BL/6 mice 6–8 weeks old, were collected and matched by age with controls. C57BL/6 mice treated with 2 cycles of 3.5% DSS for 4 days and 4 days of pure water between each cycle. After that, mice were sacrificed and the entire colon was removed. Small sections of the colon were fixed in formaldehyde, embedded in paraffin and sectioned with a microtome. Sections were stained with hematoxylin eosin to analyses the degree of inflammation.

**Results:**

After the first cycle oral administration of DSS, mice with severe and visible rectal bleeding and diarrhea entered into the acute phase. After day 4-5, bleeding and diarrhea were improved and mice entered into the chronic phase with peak levels of weight loss. Macroscopically, the inflammation was predominantly located in the distal colon. Microscopically, examination of the distal colon sections showed a decrease number of goblet cells, loss of crypts, signs of surface epithelial regeneration and moderate to severe infiltration of inflammatory cells in the mucosa.

**Conclusion:**

In order to achieve an experimental colitis model, our protocol is recommended for future therapies in IBD experimental modeling.

## Introduction

 Inflammatory bowel disease (IBD) is characterized by chronic inflammation of the gastrointestinal tract that has often its onset in patients <30 years old (15-25 years) and has a chronic relapsing-remitting course (1-3).

Despite many years of investigation, the etiopathogenesis of IBD has been remained unclear. According to the current hypothesis, the complexity of a diverse genetic, environmental such as changed gut bacteria, as well as increased intestinal permeability play an important role in dysregulation of intestinal immunity and affect the small intestine and colon.

Conventional medical management of IBD has focused on regulating the abnormal immune responses and excessive inflammation. For example, standard therapy includes sulfasalazine, 5-aminosalicylic acid, corticosteroids, immunosuppressive such as methotrexate, 6‐mercaptopurine, azathioprine, and biological agents such as infliximab (4, 5). In the case of refractory disease or specific complications such as strictureplasty, permanent colostomy or ileostomy, bowel resection surgery is an optical option (6). Recently, Helminthic therapy has been used as a potential therapeutic strategy against refractory type or complicated condition compared to conventional treatment strategies (7, 8).

The absence of parasitic infection during the childhood is an important cause for the increased prevalence of IBD (7). Animal experimentation, as well as, clinical and epidemiological studies support this theory (9, 10).

A major advance in the study of inflammatory bowel disease that provides a strong support for the above concept has been the discovery and subsequent analysis of a number of models of mucosal inflammation that resemble IBD (11, 12).

Several and variable animal models (especially mouse models) are also valuable tools to understand the pathogenesis of IBD. These models reflect the acute to chronic aspects of intestinal inflammation. IBD models fall into five main groups and each group provides opportunities to discover insights into the pathogenesis of IBD (13). These groups include: 1) Gene knockout (KO) models, including Trefoil factor-deficient mice, T cell receptor (TCR) mutant mice, Interleukin-2 KO/IL-2 receptor (R), a KO mice, IL-10 KO mice, and TNF-3’ un-translated region (UTR) KO mice. 2) Transgenic mouse models, including HLA B27 transgenic mice, IL-7 transgenic mice, Signal transducer and activating transcription (STAT)-4 transgenic mice. 3) Spontaneous colitis models including SAMP1/Yit mice, C3H/HejBir mice, cotton top tamarin. 4) Inducible models/kapten reagents, including Iodoacetamide-induced colitis, Acetic-acid-induced colitis, TNBS-induced colitis, DSS colitis, Oxazolone colitis and Peptidoglycan–polysaccharide (PG–PS) colitis. 5) adoptive transfer model including CD45RB transfer model, colitis induced by transfer of heat shock protein (hsp) 60-specific CD8 T cells.

Chemically induced mouse models of IBD are one of the most commonly used models because they are easy to induce, the onset, duration, and severity of inflammation are immediate and controllable (14,15).

The most common experimental colitis is induced by DSS. Simply changing the concentration of DSS administration can produce acute and chronic, or relapsing model. This model makes a high level of uniformity and reproducibility of most lesions in the distal colon (16, 17). 

DSS colitis in mouse model revealed several features that are found in humans, including inflammation that starts in the distal colon and then involve the proximal colon (18). A dysplasia that resembles the clinical course of human UC, occurs frequently in the chronic phase of DSS colitis (19-20). Feeding mice with 1-5% DSS dissolved in water is a cause of weight loss, shortening the intestine, mucosal ulcers, and infiltration of inflammatory granulocytes.

It is believed that DSS causes a direct hyperosmotic damage to gut epithelial cells of the basal crypts and its effects on the inner mucus layer, allowing bacteria to penetrate to this layer. Acute colitis, which occurred after feeding with DSS, was considered to be induced by innate immunity.

In susceptible strains, the administration of DSS for several cycles (e.g., 7 days DSS, 14 days water) results in chronic colitis (15, 21). Differences in resolution of disease and progression to chronicity between the C57BL/6 and BALB/c mice most likely reflect the genetic ability to mount different inflammatory responses and/or healing mechanisms as observed by the different cytokine profiles (22). In this study, we used C57BL/6 mice to create an experimental model of colitis induced by dextran sulfate sodium and investigated the correlation between the conditions of mice and the environment. 

## Materials and Methods

**Mice**

Male C57BL/6 mice 6–8 wk old, weight 17–21 g, were purchased from the animal laboratory core facility of Royan Institute of Iran. The mice were matched by age with a control group. Before starting the experiment, to rule out any parasite infection in mice, stool samples were collected from each mouse and evaluated by formalin-ether concentration.

The mice were kept in animal house facilities at the Immunology department of Shahid Beheshti University of Medical Sciences with 12:12-h light-dark cycles in standard animal cages and fed with standard pellet diet, as well as plastic bottle water ad libitum. Mice were acclimatized to these conditions for 1 week before entering the study.

**Induction of colitis**

Commercial dextran sodium sulfate (DSS) (MW 36–50kDa, MP Biomedicals, OH, USA) dissolved in a drinking water with 3.5-5% concentration (w/v) to feed C57BL/6 mice. The control group (Untreated mice) received only water. Fresh DSS solutions were prepared daily.

As C57BL/6 mice exposed to 5% DSS (for 4 days) became too sick that often happened to their death before complete the study, a lower concentration (3.5%) was used. 

For the chronic DSS-induced colitis, mice were received 2 cycles of DSS at 4 days/cycle and 4 days of pure drinking water between each cycle. In duration of adding DSS to drinking water, to control body weight, general health condition, stool consistency and fecal blood, mice monitored and data were recorded daily. 

Clinical assessment of inflammation included:

 (A) Body weight loss, negative: <1%, 1 plus:1–5%, 2 plus: 5–10%, 3 plus:10–15% and 4 plus:> 15% weight loss.

Percentage of weight loss was calculated by dividing body weight on the specified day in accordance with body weight on day 0 (baseline) (23). If each mouse had lost more than 25 percent of body weight, it was recommended to be killed by euthanasia as guidelines (24).

 (B) Stool consistency /diarrhoea. negative : normal, 1 plus: Slightly loose feces ,2 plus: loose stools, 3 plus: watery diarrhea and (C) fecal blood, negative: no bleeding, 1 plus: slightly bloody (by occult blood kit), 2 plus : bloody, 3 plus : gross bleeding and blood in whole colon . To observe the status of disposal of blood, beds of cages were covered with white paper towels that were replaced every day.

**Histology**

At the end of the procedure, mice were sacrificed and the entire colon was rapidly removed and separated from the cecum, then cleared from feces and blood by flashing cold PBS. Small sections of the colon were fixed in 4-10% formaldehyde, embedded in paraffin and sectioned at 4-5µm thickness with a microtome. Sections were stained with hematoxylin & eosin to analyse the degree of inflammation blindly (25)

The pathophysiology of the tissue was characterized by the presence of epithelial damage, inflammatory cells infiltration, crypt loss, and reduction of goblet cells. 

## Results

DSS induced models of mucosal inflammation has been used over two decades in the IBD pathogenesis and preclinical studies (26). C57BL/6 is highly susceptible to DSS colitis, but is relatively resistant to TNBS induced colitis (27).

**Figure 1:**
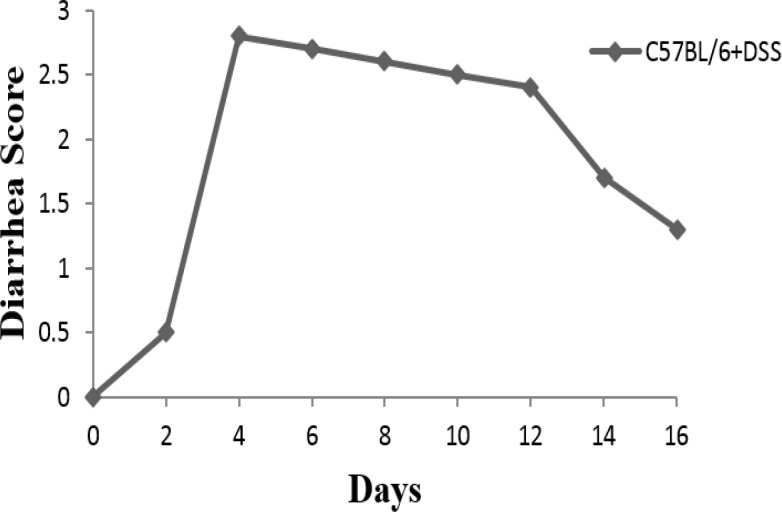
Diarrhea score in C57BL/6 mice received DSS during 16 days study

**Figure 2:**
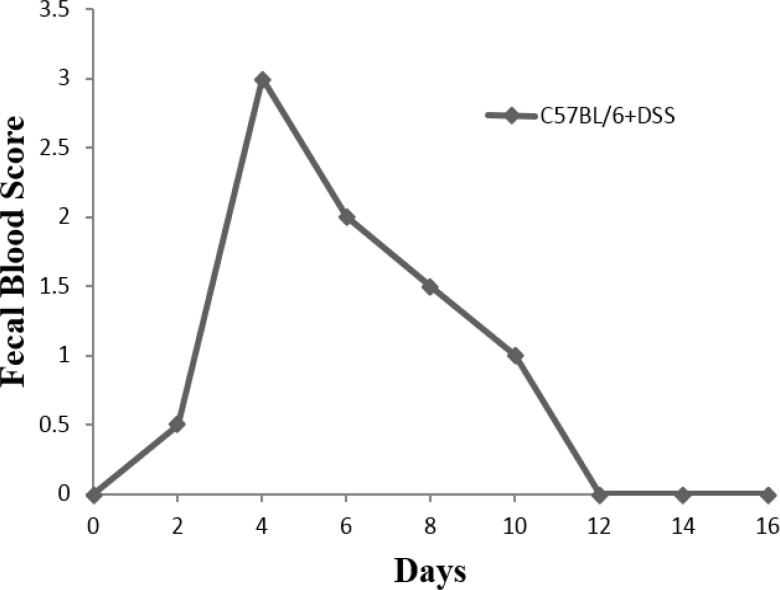
Visible fecal blood scores in C57BL/6 +DSS mice during 16 days study

**Figure 3:**
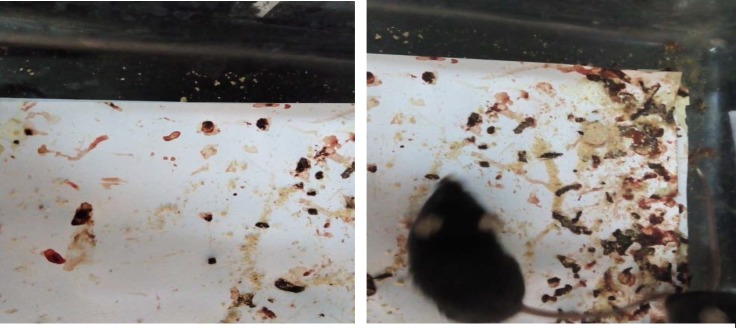
Visible bleeding and diarrhea were found on days 4-5

 After the first cycle of oral administration of DSS (first 4d), animal entered into the acute phase of disease. Visible signs of disease, including reduced mobility, a hunched back, and raised fur were seen in mice. Peak levels of sever and visible bleeding (rectal bleeding), as well as diarrhea were found at day 4 (Figures 1, 2, and 3).

**Figure 4:**
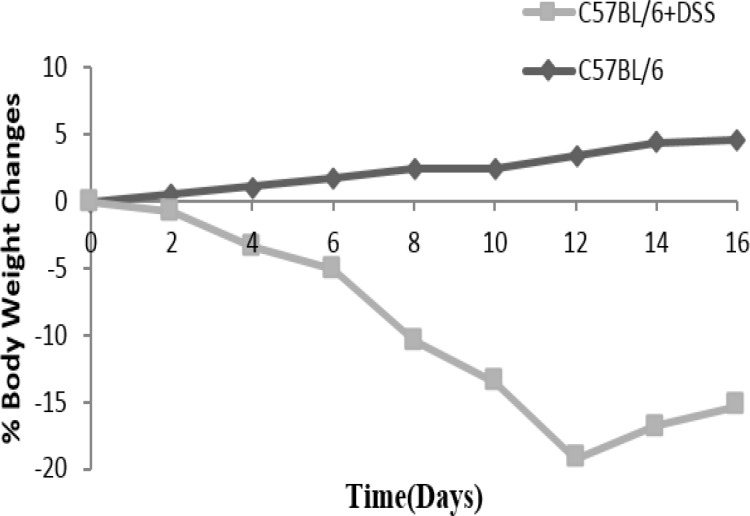
Daily changes in body weight in C57BL/6 mice received pure water compared with C57BL/6+DSS mice

After day 4-5, improvement of bleeding and diarrhea were seen and mice entered into the chronic phase. Weight loss was started from day 4 in C57BL/6 mice (Figure 4).

Body weights were increased a little during the first three days and began to decrease while the bleeding started.

Macroscopically, the inflammation was predominantly located in the distal colon. The length of the colon reduced as the disease developed and was significantly shorter at day 4 (18-22%).

**Figure 5:**
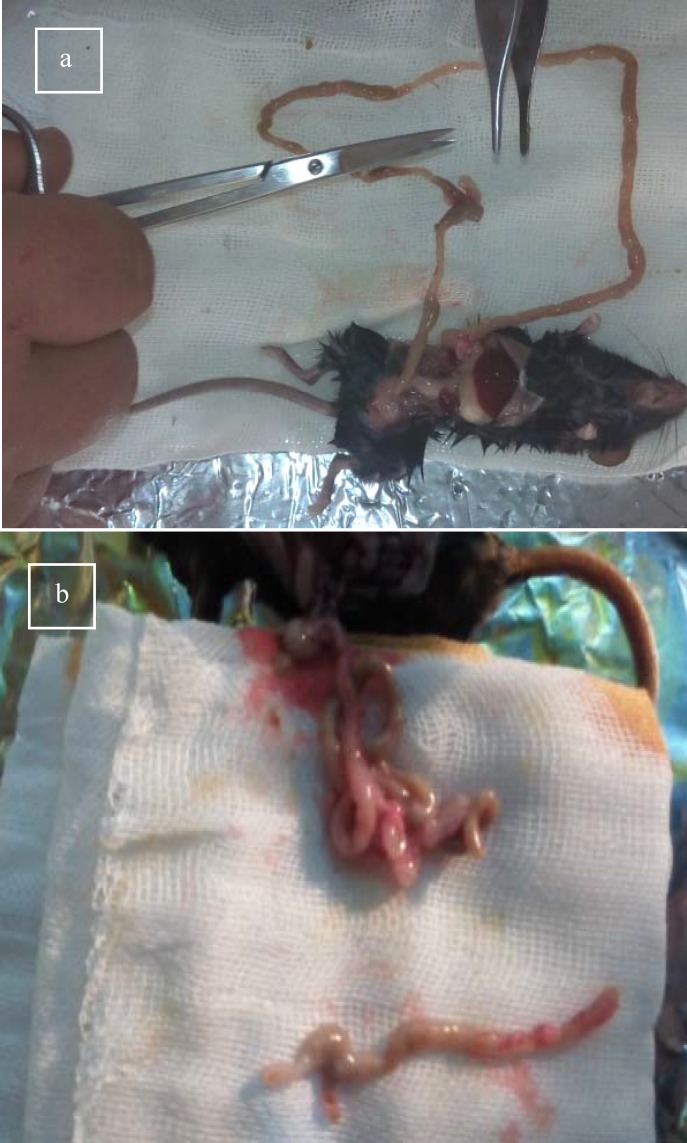
Grossly thickening of the colon wall in DSS colitis was observed .a) In all parts of intestine. b) Colon segment.

Reduction in cecum size, as well as occasional blood in the small intestinal lumen, and stomach was observed on day 4. Furthermore, thickening of the colon wall in DSS colitis was observed (Figure 5).

Oral administration of DSS in 2 cycles was induced chronic colitis in C57BL/6. Chronic colitis was characterized by a noticeable decrease in colon length, bleeding, and ulceration in mouse models, and grossly thickened walls.

**Figure 6:**
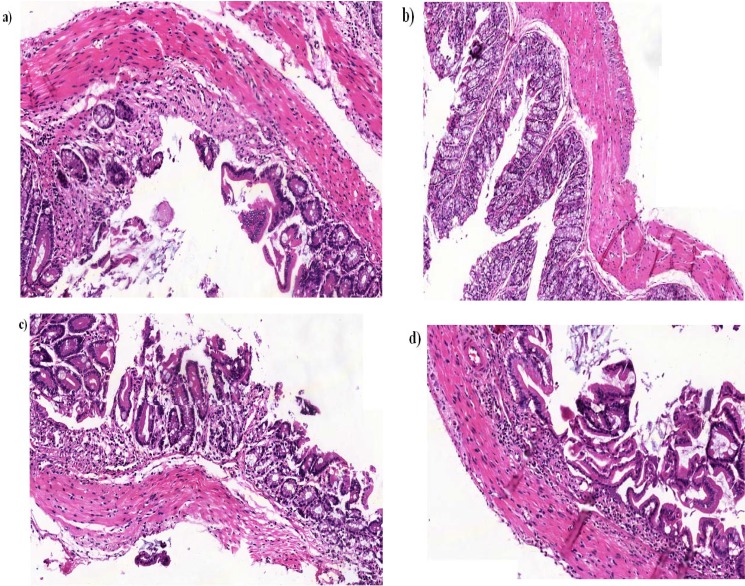
Consideration of histopathologic changes in the mice that received 2 cycles of DSS for 4 days and 4 days pure water between each cycle. a) Loss of crypts; b) Slightly changes in the surface epithelium; c) Reduction of goblet cells; d) Visible infiltration of inflammatory cells

**Pathological analysis**

Microscopic examination of the distal colon sections stained by hematoxylin and eosin showed a marked inflammation in C57BL/6 mice treated with 2 cycles of DSS in 4 days/cycle during 16 days study.

C57BL/6 +DSS mice showed small changes in the surface of the epithelium, although a visible infiltration of inflammatory cells to the mucosa was seen.

The histological changes were clearly shown by a decreased number of goblet cells and loss of crypts, signs of surface epithelial regeneration, as well as moderate to severe infiltration of inflammatory cells to the mucosa (Figure 6). 

## Discussion

Different mouse models have been greatly used to understand the pathophysiology and developing therapeutic strategies for IBD (28). DSS induced colitis model is very popular in IBD research due to its simplicity, reproducibility, rapidity and controllability. The DSS-induced colitis model gives very important information regarding the interactions between gut immunity, microbiota, host genetics, diet and other environmental factors in maintaining gastrointestinal homeostasis (24).

In this study, we described a new approach to induce progressive chronic colitis in C57BL/6 mice. We also showed different phases of colitis development, which classified as acute, chronic, and recovery phases. This classification is based on the clinical symptoms and histopathological changes. 

DSS is often administered in a dose range of 1–5% for 7-14 days to induce an acute IBD in C57BL/6 mice (21, 29). To induce chronic phase of IBD, DSS is administered in three to five cycles with a 1- 2 week rest period between cycles (15). Our results showed that 2 cycles of 3.5% DSS for 4 days and 4 days of pure drinking water between each cycle is sufficient to induce chronic phase of IBD in C57BL/6 mice. We have seen that the acute phase (day 4) was characterized by clinical symptoms (visible rectal bleeding, diarrhea, and weight loss), and chronic phase (day 12 and after that) was characterized by recovering body weight, few clinical symptoms, high inflammatory score, and histopathological changes.

We examined that exposer to 4-5% DSS (for 4 days) has often led to the death of mice; therefore lower concentration (3.5%) was used. Based on clinical and histopathological findings, our results were similar to other articles (15,21,29). However, we achieved very acceptable results in less time. Also, low doses of DSS (1-2 %) were tested in our study but did not achieve the satisfactory results. The mice at day 7 were entered in the acute phase, but did not have a good physical condition and most of them died on day 10.

Wirtz et al. recommended that three to five cycles with one week interval is needed for chemically induced mouse models of intestinal inflammation, while noticing that at the end of the second cycle, the weight has been 20% decreased and continuing other cycles may lead to the death of animals.

The main difference between induced DSS colitis procedures model in this study and other investigations are confounding factors in the experimental setup, such as environmental conditions (intestinal micribiota, pathogen infection, food composition, stress factors, light), mouse house origin, and genetic backgrounds (30).

In summary, the acute and chronic model of colitis in C57BL/6 mice may be useful in the study of pathological inflammatory changes that were observed in ulcerative colitis. Also, we suggest that the chronic model is a useful and powerful tool that can be used to validate future therapeutic strategies and candidate treatments for IBD. Inducing experimental IBD model by chemical materials, mostly DSS, should be considered according to the environmental conditions and microbial flora differences.
